# Haploinsufficiency of DNA Damage Response Genes and their Potential Influence in Human Genomic Disorders

**DOI:** 10.2174/138920208784340795

**Published:** 2008-05

**Authors:** Mark O’Driscoll

**Affiliations:** Genome Damage & Stability Centre, University of Sussex, Falmer, Brighton, East Sussex, BN1 9RQ, UK

**Keywords:** DNA damage response, ATR, haploinsufficiency, genomic disorders.

## Abstract

Genomic disorders are a clinically diverse group of conditions caused by gain, loss or re-orientation of a genomic region containing dosage-sensitive genes. One class of genomic disorder is caused by hemizygous deletions resulting in haploinsufficiency of a single or, more usually, several genes. For example, the heterozygous contiguous gene deletion on chromosome 22q11.2 causing DiGeorge syndrome involves at least 20-30 genes. Determining how the copy number variation (CNV) affects human variation and contributes to the aetiology and progression of various genomic disorders represents important questions for the future. Here, I will discuss the functional significance of one form of CNV, haploinsufficiency (i.e. loss of a gene copy), of DNA damage response components and its association with certain genomic disorders. There is increasing evidence that haploinsufficiency for certain genes encoding key players in the cells response to DNA damage, particularly those of the Ataxia Telangiectasia and Rad3-related (ATR)-pathway, has a functional impact. I will review this evidence and present examples of some well known clinically similar genomic disorders that have recently been shown to be defective in the ATR-dependent DNA damage response. Finally, I will discuss the potential implications of a haploinsufficiency-induced defective DNA damage response for the clinical management of certain human genomic disorders.

## 1 INTRODUCTION

It has long been appreciated that changes in gene copy number are associated with phenotypes in humans. Perhaps, the most well known example of this is the trisomy 21 causative of Down syndrome. Here, increased expression of the genes on chromosome 21 results directly or indirectly in a clinically heterogeneous disorder incorporating cognitive impairment, facial dysmorphology, growth retardation, cancer predisposition, microcephaly, heart and skeletal abnormalities [1]. Interestingly, it was recently shown that CNV is in fact a common genetic trait in clinically unaffected or ‘normal’ individuals [2]. Indeed, the first complete genomic sequence from an individual, the so-called ‘Venter genome’ yielded a surprising level of CNV, highlighting the plasticity of the human genome [3]. The phenomenal recent revolution in the sensitivity and widespread usage of array-based Comparative Genomic Hybridization (array-CGH/a-CGH) techniques has led to CNV being described as the ‘Breakthrough of the Year’ by the journal Science [4]. The widespread use of a-CGH has facilitated the description of several novel genomic disorders and aided in the detailed genetic characterisation of known genomic disorders. A persistent challenge to clinical geneticists and researchers is to unravel exactly how changes in CNV of specific genes or various combinations of genes can impact on normal development. These issues have recently been extensively reviewed elsewhere and the reader is directed to these sources for an overview of a-CGH technology and its applications [5-12]. Here, I will discuss the potential role of haploinsufficiency of DNA damage response components, particularly that of the ATR-pathway, in genomic disorders. Contiguous gene deletion disorders represent a clinically diverse group of human genomic disorders caused by distinct heterozygous chromosomal deletions usually involving several genes [5, 13]. It is assumed that the various clinical manifestations of different genomic disorders arise from the combined impact of haploinsufficiency of multiple genes [14]. It is likely that there are critical genes or pathways sensitive to haploinsufficiency either alone or when combined with haploinsufficiency of other genes. There is increasing evidence from both human and murine studies suggestive of a cellular impact of haploinsufficiency of genes that control different aspects of the response to DNA damage. Since our genomes are constantly exposed to exogenously-derived (e.g. UV radiation) and endogenously-derived (e.g. metabolically generated reactive oxygen species) DNA damaging agents, an impaired ability to detect and/or respond appropriately to these threats can impact on the maintenance of genetic stability. There are many examples of human Mendelian disorders defective in the repair of or response to DNA damage [15]. The importance of these pathways is demonstrated by the increase in cancer predisposition and developmental abnormalities associated with these conditions [15]. An important DNA damage response (DDR) pathway that appears to be affected by haploinsufficiency is the ATR-dependent DDR (ATR-DDR).

## 2 THE ATR-DEPENDENT DNA DAMAGE RESPONSE (ATR-DDR)

The DDR can be divided into DNA repair processes and signal transduction processes that sense DNA damage and co-ordinate the appropriate response such as cell cycle checkpoint activation, DNA repair and/or apoptosis. Two phosphoinositol-3-kinase-like protein kinases (PIKK) coordinate the signal transduction response to DNA damage in mammalian cells [16-18]. Ataxia Telangiectasia Mutated (ATM) is activated following DNA double strand breaks (DSBs) whilst a related kinase, Ataxia Telangiectasia and Rad3-related (ATR), responds to single stranded regions of DNA. Single stranded DNA (ssDNA) can act as an intermediate during the activity of several excision-mediated repair pathways (nucleotide excision repair and base excision repair), during uncoupling of the transcriptional machinery from its DNA template or similarly following stalling of the DNA replication machinery at DNA lesions or single strand breaks. Following the exposure of ssDNA, it is quickly coated by RPA, a heterotrimeric protein complex (RPA1-3) that plays a role in many aspects of DNA metabolism (e.g. replication, repair, transcription). ATR is recruited to the ssDNA *via *its binding partner ATRIP (ATR Interacting Protein) [19, 20]. Topoisomerase binding protein 1 (TopBP1) appears to be required for optimal ATR kinase activity [21]. Phosphorylation of the histone H2A variant H2AX on Ser-139 (called γ-H2AX) is one of the earliest detectable PIKK-dependent responses to DNA damage and is required for the retention of additional damage response proteins at the damage site [22-24]. The Mre11/Rad50/NBS1 complex, the Rad17/Rfc2-5 and Rad9/Rad1/Hus1 complexes are recruited to the site of damage independently of ATR/ATRIP and are also phosphorylated by ATR [25, 26]. Retention of these complexes facilitates ATR’s ability to phosphorylate downstream substrates including Brca1, p53 and its effector kinase Chk1 [15].

There is a large amount of functional overlap between ATM and ATR. In fact, both kinase’s phosphorylate mainly the same substrates in response to DNA damage (e.g. Mre11/Rad50/NBS1 complex, p53, Brca1). DSBs can undergo exonucleolytic resection generating ssDNA overhangs, hence generating an ATR activating substrate [27]. Conversely, ssDNA generated at stalled replication forks can collapse producing overt DSBs, hence generating an ATM activating substrate [28]. Nevertheless, congenital defects in ATM and ATR are associated with clinically distinct human disorders. Mutations in *ATM *cause Ataxia telangiectasia, a progressive degenerative neurological disorder characterised by ataxia and lymphoid malignancy predisposition [29, 30]. Mutations in *ATR *result in Seckel syndrome, a disorder characterised by microcephaly and growth retardation [31, 32].

## 3 ATR AND GENOMIC STABILITY

ATR is required to maintain genomic stability. Whilst it has a role in the stabilisation of stalled replication forks, the absence of ATR results in a very specific type of genomic instability, namely DNA Fragile Site expression. DNA Fragile Sites (DFS) are large (>100Kb) distinct genomic regions that exhibit breaks under conditions of replicative ‘stress’ [33, 34]. Such stresses can be induced in the laboratory using DNA replication inhibitors such as aphidicolin or by folate deficiency, which can indirectly impact on the availability of dNTP’s. There are about 75-80 DFS throughout the human genome but it is not really clear why DFS are so unstable. Most tend to be relatively AT-rich and contain more areas of flexibility than non-fragile site regions. Studies on the replication timing of certain fragile sites (FRA3B, FRA7H, FRAXA on chromosome 3, 7 and X respectively) indicate that they are replicated very slowly [33]. DFS are ‘hot spots’ for sister chromatid exchanges (SCE’s) and are also thought to play a role in gene amplification events *via *a breakage-fusion-bridge cycle [33]. Breakage at or ‘expression of’ DFS is associated with many cancers. For example, the *FHIT *tumour suppressor gene spans the DFS FRA3B [35]. This gene is often re-arranged or partially deleted in a wide range of tumours (lung, ovarian, breast, esophageal) [36]. Pioneering work by the Glover laboratory identified ATR as the first protein required to mediate DFS stability [37]. Subsequently, several other proteins have been implicated in maintaining DFS stability including the Brca1, SMC1, WRN helicase, Chk1 and the Fanconi anaemia pathway components FANC-A, FANC-B and FANC-D2 [33, 38-41]. Interestingly, most of these proteins are known direct ATR substrates.

## 4 SECKEL SYNDROME: AN ATR-PATHWAY DEFECTIVE DISORDER

Seckel syndrome, originally described as ‘Bird headed Dwarfism’ in a 1960 monograph by Dr. Helmut Seckel, is a disorder characterised by severe microcephaly, isolated skeletal abnormalities (clinodactyly, thoracic kyphosis, ivory epiphysis) and a dramatic proportionate primordial dwarfism [42]. Microcephaly is a clinical term describing a reduction in occipitofrontal (or head) circumference greater than 3 standard deviations (-3 s.d.) below the age-related mean. This reduced head circumference is a consequence of premature closure and fusion of the cranial sutures reflecting the underlying reduction in brain volume. The aetiology of microcephaly is complex. It can occur in the context of genetic (e.g. syndromal) or non-genetic (e.g. intrauterine infection) situations. Microcephaly is particularly pronounced in Seckel syndrome [31, 42, 43]. The first genetic defect associated with Seckel syndrome was described in 2003. A single synonymous hypomorphic mutation in *ATR* was identified in five affected individuals in two consanguineous Pakistani families [31]. The mutation (A21201G) was shown to adversely impact on splicing, resulting in dramatically reduced ATR expression in cells derived from the affected patients. Gene targeting of *ATR* in the murine system results in early embryonic lethality [44, 45]. Hence, these ATR-defective Seckel syndrome cells (ATR-S) proved a useful tractable model to investigate ATR-pathway function in the mammalian setting. ATR-S cells exhibited a diminished ability to phosphorylate ATR substrates following DNA damage-induced ATR-pathway activation (e.g. γH2AX formation and p53-serine-15 phosphorylation) as well as defective cell cycle checkpoint arrest and increased DFS expression [31, 46, 47].

Seckel syndrome is known to be genetically heterogeneous [43, 46, 48-50]. Interestingly, Alderton *et al. *showed that several unrelated non-*ATR *mutated Seckel syndrome cell lines all exhibited defective ATR-pathway function (e.g. failure of ATR-dependent G2-M cell cycle checkpoint arrest) [46]. Hence, in agreement with the known genetic heterogeneity of this condition, whilst not all Seckel syndrome cases are caused by mutations in *ATR* itself, interestingly, all Seckel syndrome cell lines (ATR-S and non-ATR-S) exhibit compromised ATR-pathway function [46]. A further interesting cellular feature of ATR-pathway defective Seckel cells is supernumerary centrosomes in 10-20% of mitotic cells [46]. A normal mitotic cell must only have two centrosomes that nucleate the microtubule spindles facilitating transfer of an equal and identical chromosome complement to the daughter cells [51, 52]. The molecular aetiology of supernumerary mitotic centrosomes in Seckel syndrome cells is currently unclear. Centrosome orientation is fundamental for determining symmetric and asymmetric stem cell division in the embryonic neuroepithelium, an essential process for normal brain development [53-55]. Hence, this cellular feature may be a relevant contributor to the severe microcephaly characteristic of Seckel syndrome. Recently, the second genetic defect associated with Seckel syndrome was described. Mutations in *pericentrin (PCNT2)/kendrin* which encodes a structural centrosomal protein, were identified in several Seckel syndrome patients all of which exhibited defective ATR-pathway function [56]. This exciting finding further illustrates a functional link between the DDR, the cell cycle machinery and the centrosome. Furthermore, mutations in *PCNT2 *were also recently found in several microcephalic osteodysplastic primordial dwarfism type II (MOPD II) patients [57, 58]. Whether *PCNT2*-mutated Seckel syndrome and MOPD II represent distinct disorders or allelic variants of the same condition remains an open question.

## 5 OTHER DISORDERS THAT EXHIBIT COMPROMISED ATR-PATHWAY FUNCTION

Compromised ATR-pathway function does not appear to be uniquely associated with Seckel syndrome. Several other known DDR disorders have been shown to be defective in aspects of ATR-pathway activity, particularly cell cycle checkpoint activation. Interestingly, whilst these disorders are all characterised by a distinct set of clinical features, there is significant clinical overlap with Seckel syndrome, particularly concerning the developmental abnormalities such as microcephaly and growth retardation (Table **1**).

### 5.1 Nijmegen Breakage Syndrome (NBS)

NBS is caused by hypomorphic mutations in the *Nbs1 *component of the Mre11/Rad50/NBS (MRN) complex [59, 60]. The MRN complex plays a central role in ATM signalling where it is thought to recruit ATM to the DSB and also facilitate its ability to phosphorylate substrates such as p53 and Chk2 [61]. In fact, all the components of this complex are ATM substrates. Historically, NBS has been described as an A-T-like disorder as cell lines from both conditions exhibit radio-sensitivity and similar cell cycle checkpoint defects in response to DSBs. But, NBS1 is also an ATR substrate and NBS patients exhibit microcephaly and growth retardation, clinical features associated with Seckel syndrome and not A-T. Recently, NBS cells were shown to be compromised for ATR-dependent checkpoint activation and other aspects of ATR-pathway function [26]. Therefore, NBS represents a human condition defective in elements of both ATM and ATR-pathway activity.

### 5.2 Fanconi Anaemia (FA)

FA, is a genetically heterogeneous condition characterised by a progressive aplastic anaemia, skeletal abnormalities, microcephaly and lymphoid malignancy. FA is caused by mutations in different genes whose products co-ordinately function in the cellular response to DNA cross-links [62, 63]. Several of these genes encode products that together mediate the monoubiquitylation and activation of the FANC-D2 protein in response to DNA damage, particularly during S phase [62]. ATR and NBS1 have been shown to be required for this specific modification [64-66]. In fact, ATR-S and NBS patient-derived cell lines fail to monoubiquitylate FANC-D2 following treatment with replication fork inhibitors [26]. In addition to undergoing monoubiquitylation, FANCD2 has been shown to be phosphorylated by ATM and ATR further highlighting the over lap between these DDR pathways [26, 64, 67].

### 5.3 MCPH1-Dependent Primary Microcephaly

Autosomal recessive Primary Microcephaly, clinically characterised by the presence of a severe microcephaly in the absence of other overt clinical features, is a genetically heterogeneous condition composed of six distinct genetic complementation groups. To date, mutations in four genes, all of which encode centrosomal proteins, have been described for this disorder (*MCPH1, ASPM, CDK5RAP2, CENPJ*) [55, 68-71]. *Microcephalin (MCPH1)*, the first Primary Microcephaly gene identified encodes a BRCT-containing product that has been implicated in the response to DSBs [72, 73]. Importantly, work using MCPH1-patient derived cell lines with hypomorphic mutations in *MCPH1* indicated that these cells are defective in ATR-dependent checkpoint activation and also exhibit supernumerary mitotic centrosomes [74]. Furthermore, MCPH1 was shown to interact with Chk1, a substrate and downstream effector of ATR [74].

## 6 THE IMPACT OF *ATR* HAPLOINSUFFICIENCY: MURINE STUDIES

All of the disorders described so far that collectively exhibit defective ATR-pathway function are all caused by gene mutations inherited in an autosomal recessive manner. As mentioned earlier, a knockout mouse model for ATR does not exist due to early embryonic lethality of ATR^-/-^ blastocysts [44, 45]. Whilst ATR^+/-^ animals are viable, re-evaluation of this murine work suggests that the ATR-DDR pathway is sensitive to gene copy number variation. Firstly, ATR^+/-^ mice are not born at the expected Mendelian frequency suggesting a requirement for a full diploid complement of ATR protein during normal development [75]. Furthermore, decreased survival and increased tumour incidence were recorded in the ATR^+/-^ animals compared to their ATR^+/+^ counterparts [75]. This is distinct to that of ATM^+/- ^ embryos and mice. Heterozygous mutations in *ATR * have been observed in microsatellite unstable human colorectal carcinomas further suggesting that *ATR* haploinsufficiency may play a role in tumourigenesis [76]. In fact, it has been suggested that ATR acts as a haploinsufficient tumour suppressor under certain circumstances. Fang and colleagues showed that ATR^+/-^ mice when crossed into a mismatch repair defective (Mlh1^-/-^) background (generating ATR^+/-^/Mlh1^-/-^) were highly susceptible to embryonic lethality and premature tumour development [77].

## 7 THE IMPACT OF *ATR* HAPLOINSUFFICIENCY: HUMAN STUDIES, INCLUDING THE ASSOCIATION OF ATR^+/-^ WITH A GENOMIC DISORDER

Fang and colleagues also showed that haploinsufficiency of *ATR* in human cells results in a clear DDR-defective phenotype [77]. Following gene targeting of *ATR* in the human HCT116 colorectal carcinoma cell system, they found a significantly increased expression of DFS as well as other gross chromosomal rearrangements and amplifications. Furthermore, these ATR^+/- ^cells exhibited diminished ATR-mediated phosphorylation of its effector kinase Chk1.

Work from de Ru and colleagues along with that of O’Driscoll and colleagues has provided evidence for a cellular impact of ATR haploinsufficiency associated with a human genomic disorder [78, 79]. Blepharophimosis-ptosis-epicanthus inversus syndrome (BPES) is a disorder characterised by a reduction in the dimensions of the palpebral fissures or eye sockets (blepharophimosis), drooping eyelids (ptosis) and inverted skin folds originating from the lower eyelids (epicanthus inversus) [80]. A variant of this disorder is also associated with ovarian failure and female infertility [81]. This disorder is caused by autosomal dominant mutations in or heterozygous deletion of *FOXL2,* a putative forkhead transcription factor [80]. In a review of the literature, de Ru and colleagues noted that most of the BPES patients with a cytologically detectable deletion or a microdeletion on chromosome 3q, where *FOXL2* resides, also exhibited microcephaly and short stature [78]. These are clinical features not typically associated with ‘classical’ BPES. Indeed, it had been previously proposed that a putative gene for microcephaly was located close to the BPES-causative gene on chromosome 3q (reviewed in [78]). De Ru and colleagues mapped the heterozygous deletion in one such BPES patient with microcephaly and short stature. They found that this patient was haploinsufficient for both *FOXL2 *and *ATR* at the genomic level. They suggested that the haploinsufficiency of *ATR* may be responsible for the microcephaly and short stature observed in this patient based on the occurrence of these clinical features in ATR-defective Seckel syndrome [78]. In a complementary study, O’Driscoll and colleagues subsequently showed that cells from this BPES-ATR^+/-^ patient exhibited similar cellular defects to ATR-S cells [79]. These cells failed to show significant γH2AX formation and Chk1 phosphorylation following replication fork stalling. They also exhibited a similar ATR-dependent G2-M cell cycle checkpoint defect to ATR-S cells. Importantly, this phenotype was corrected following over-expression of *ATR* in the BPES-ATR^+/-^ cells. This study reinforced the fact that haploinsufficiency of *ATR *has a functional impact in human cells but furthermore, that haploinsufficiency of *ATR* is associated with a human genomic disorder that exhibits microcephaly and short stature [79].

## 8 HAPLOINSUFFICIENCY OF ATR-PATHWAY COMPONENTS IN OTHER GENOMIC DISORDERS

Work from Lam and colleagues using tissue-specific conditional knockdown in mice of Chk1, an important effector kinase and substrate of ATR, showed that Chk1 haploinsufficiency enhances mammary tumourigenesis [82]. This work suggested that an ATR-pathway component and not just ATR itself is sensitive to haploinsufficiency. Following the cellular characterisation of BPES- ATR^+/-^, working on the presumption that the ATR-pathway as a whole is sensitive to a reduced gene copy number, O’Driscoll and colleagues examined ATR-pathway function in other human genomic disorders whose causative genomic deletions were known to result in haploinsufficiency of various ATR-pathway components [79] (Table **2**).

### 8.1 Isolated Lissencephaly Sequence (ILS) and Miller-Dieker Lissencephaly Syndrome (MDLS)

**Fig. (1):**
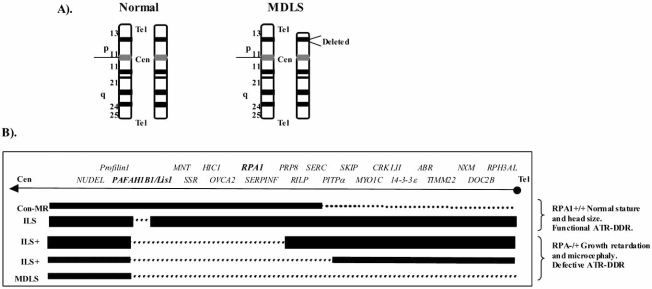
**A)**. Chromosome 17 karyotype from a normal and MDLS individual. **B)**. A view of a single chromatid of chromosome 17p13.3 showing the order of the genes in this region from the centromere (Cen) to the telomere (Tel). The dotted lines denote the deletions (hemizygous) of various sizes associated with respective patients listed on the left hand side. Con-MR is a patient with a telomeric deletion. This patient exhibits mental retardation but normal stature and head circumference. ILS represents an Isolated Lissencephaly Sequence patient deleted for PAFAH1B1/Lis1 alone. ILS+ denotes Isolated Lissencephaly Sequence patients with increasing deletions telomeric from PAFAH1B1/Lis1. MDLS represents Miller-Dieker Lissencephaly.

Normal human brain development involves a rapid and sustained cellular proliferation originating from the rostral end of the foetal neural tube. Cerebral cortical development is achieved *via *a highly regulated sequence of neuroprogenitor cell division, migration and differentiation. Platelet-activating factor acetylhydrolase, isoform B1 *(PAHFAH1B1/Lis1),* located on human chromosome 17p13.3, encodes a protein, Lissencephaly 1 (Lis1), which plays a central role in neuronal migration from the neuroepithelial stem cell layer during embryonic brain development [83-85]. Mutations or heterozygous deletions of *PAHFAH1B1/ Lis1 *alone causes Isolated Lissencephaly Sequence (ILS), a disorder characterised by reduced neuronal migration resulting in a cortical surface without significant invaginations, effectively a "smooth brain" (lissencephaly) (Fig. **1**). Larger deletions identified in some ILS patients confer a more severe grade of lissencephaly associated with additional craniofacial abnormalities (ILS+) (Fig. **1**). Even larger deletions extending telomerically from the *PAHFAH1B1/Lis1 *gene are associated with Miller-Dieker Lissencephaly Syndrome (MDLS), which is characterised by the most severe grade of lissencephaly, craniofacial abnormalities, microcephaly and growth retardation (Fig. **1**) [14, 86]. Unlike ILS patients, ILS+ patients and all MDLS patients are haploinsufficient for *RPA1*, the largest subunit of the Replication Protein A complex (Fig. **1**) [86]. As described previously, this complex coats ssDNA generated for example at a stalled replication fork allowing the recruitment and ultimate activation of ATR [19]. Similar to what was observed using BPES-ATR^+/- ^cells, cell lines from ILS+ and MDLS patients collectively exhibited compromised ATR-pathway function [79]. Furthermore, these cellular defects could be complemented following over-expression of *RPA1* in these cells. ILS patient cell lines with heterozygous deletions in *PAHFAH1B1/ Lis1* only, by contrast, exhibited a functional ATR-pathway response (Fig. **1**). Therefore, ILS+ and MDLS represent two further human genomic disorders with a clinical overlap with ATR-S that also exhibits compromised ATR-pathway function at the cellular level [79].

### 8.2 Williams-Beuren Syndrome (WBS)

WBS is caused by a hemizygous sub-microscopic deletion of 1.55-1.84Mbp on chromosome 7q11.23 encompassing around 25-30 genes. The clinical presentation of this condition is multifaceted including craniofacial, endocrinological and cardiovascular abnormalities along with microcephaly and growth retardation [87, 88]. One of the genes heterozygously deleted in WBS is *ELN*, which encodes elastin, a key structural component of vascular tissues. A characteristic cardiovascular abnormality of WBS, supravalvular aortic stenosis (narrowing of the ascending aorta), is thought to specifically derive from haploinsufficiency of *ELN* [89]. WBS patients are also haploinsufficient for *RFC2 *a gene that encodes a component of the Replication Factor C (RFC) complex that plays a fundamental role during DNA replication loading PCNA onto chromatin, thus facilitating DNA polymerase action [90]. RFC2 is also a component of the Rad17-RFC2-5 complex that is known to function in the DDR [91-94]. In fact, Rad17 is phosphorylated by ATM and ATR following DNA damage. Interestingly, a yeast stain (*S. cerevisae*) with a hypomorphic mutation in *Rfc2* fails to activate cell cycle arrest following DNA damage, suggesting that the RFC complex and specifically *RFC2* plays a direct role in the DDR [95]. O’Driscoll and colleagues also showed that WBS patient-derived cell lines exhibit a defective ATR-dependent DDR that could be complemented following reintroduction of *RFC2* into these cells [79]. Therefore, WBS represents another genomic disorder that exhibits defective ATR-pathway activity wherein microcephaly and growth retardation are included in its clinical spectrum [79].

Of course, for all of the genomic disorders discussed above that exhibit a defective ATR-dependent DDR associated with microcephaly and short stature, the occurrence of this specific DDR defect with these particular clinical features is associative. This association, albeit strong, remains an association only. Hence, more work using complementary systems such as gene targeting or tissue-specific knockdown in the murine system will be required to definitively prove the link between ATR-pathway dysfunction and these developmental abnormalities.

## 9 HAPLOINSUFFICIENCY OF OTHER DDR COMPONENTS

Increasing evidence from murine gene targeting studies suggests that haploinsufficiency of various components of a diverse distinct range DDR pathways and not just *ATR* or ATR-pathway components has a functional impact on maintaining genomic stability. For example, haploinsufficiency of various components of the mitotic spindle checkpoint such *BubR1* or *MAD2 *is associated with aberrant chromosome segregation and aneuploidy in mice [96, 97]. Recently, haploinsufficiency of another mitotic spindle checkpoint component, *Mad1*, has been shown to be associated with aneuploidy and increased constitutive tumour incidence in *Mad1^+/-^* mice compared to their wild-type (*Mad1^+/+^*) littermates [98]. Homologous recombination (HR) is an error-free DSB repair pathway used by mammalian cells when a homologous sister chromatid is available as a template for DNA repair (S- and G2-phases of the cell cycle) [99]. Haploinsufficiency of multiple genes whose products function in HR results in compromised genomic stability. Specific examples include *Brca1^+/-^, Xrcc2^+/-^, Xrcc3^+/-^, Rad51b^+/-^* and *Rad51d^+/-^* mice, all of which present with recombination deficiency, increased chromosomal aberrations and centrosomal fragmentation [100-105]. The Mus81-Eme1 complex functions as a structure specific endonuclease that plays a role in resolving stalling replication forks, 3’-orientated DNA flaps/overhangs and nicked HR intermediates [106]. Haploinsufficiency of both components of the structure specific endonuclease Mus81-Eme1 results in increased chromosomal aberrations and a re-replication phenotype in human and murine cells [107]. Poly(ADP-ribose) polymerase-1 (PARP-1) catalyzes the covalent attachment of long branched poly(ADP-ribose) polymers onto a diverse set of target proteins (including itself), using NAD+ as its substrate. Attachment of these negatively charged polymers changes the biological activity and properties of the target proteins. PARP1 plays an important role in sensing single stranded breaks in DNA [108-110]. The level of PARP activity is sensitive to PARP-1 gene dosage [111]. Interestingly, PARP1^-/+^ mouse embryonic fibroblasts were shown to exhibit increased supernumerary centrosomes relative to their PARP^+/+^ counterparts [111]. Furthermore, haploinsufficiency of the histone H2A sub-family member *H2AX*, a known ATM and ATR substrate, has been shown to compromise genomic integrity, impact on the normal response to DNA damage and enhance tumour susceptibility in the absence of *p53* in mice [112].

## 10 IMPLICATIONS OF HAPLOINSUFFICIENCY OF DDR COMPONENTS IN HUMAN GENOMIC DISORDERS

Murine gene targeting studies have proved invaluable in identifying DDR pathways that are sensitive to haploinsufficiency (e.g. the spindle checkpoint and HR). It is likely that haploinsufficiency of these pathways will potentially contribute to the clinical features of known and/or novel genomic disorders. A major consequence of compromised DDR is increased genomic instability and cancer predisposition (reviewed in [15]). Additionally, since DNA damaging agents are the cornerstone of clinically utilised therapeutic approaches for cancer, individuals with compromised DDR are hypersensitive to such treatments. This has been observed in Ataxia telangiectasia, Nijmegen breakage syndrome, LIG4 syndrome and Fanconi anaemia patients, some of which have fatally over-responded to standard radio- and/or chemotherapy regimens in the past (reviewed in [113]). Since increased life expectancy due to improved medical supervision is now a feature of many genomic disorders, a potentially defective DDR may become more important from the perspective of tumour development and treatment. This could be particularly relevant for genomic disorders with compromised ATR-pathway function. Whilst it is not clear whether conditions such as BPES-ATR^+/-^, MDLS and WBS represent tumour-predisposition conditions, it is clear that they do exhibit a defective ATR-dependent DDR [79]. Provocatively, isolated reports of malignancy in these disorders, particularly in WBS exit  [114-117]. Whether a compromised ATR-dependent DDR has a role here is currently unclear although worthy of deeper investigation.

In conclusion, plasticity of the human genome is reflected in the high level of CNV observed in clinically normal individuals. Nevertheless, CNV is causative of many pathological conditions in humans. Gene-targeting studies in mice have shown that one form of CNV, namely haploinsufficiency, of certain DDR-pathway components is associated with compromised genomic stability. Haploinsufficiency of ATR, or some of its pathway components confers similar DDR defects to that of ATR-pathway defective Seckel syndrome cell lines. Furthermore, haploinsufficiency of *ATR, RPA1* and *RFC2* is associated with several human genomic disorders that exhibit microcephaly and growth retardation. Haploinsufficiency of DDR pathway components is likely to contribute to the clinical features of many genomic disorders. This will have implications for the clinical management and treatment of these conditions.

## WEB RESOURCES

Online Mendelian Inheritance in Man, (http://www.ncbi.nlm.nih.gov/Omim), for reviews of the specific genes and all of the disorders described here.

DECIPHER, (https://decipher.sanger.ac.uk/), a database collating multiple genomic imbalances and their associated clinical features in human genomic disorders.

EUCARUCA, (http://agserver01.azn.nl:8080/ecaruca/whatisEc.jsp), a database of cytogenetic and clinical data of rare chromosomal aberrations from all centres that are member of the European Cytogeneticists Association (ECA).

Database of Genomic Variants, (http://projects.tcag.ca/variation/), a database listing a comprehensive summary of structural variation in the human genome.

## Figures and Tables

**Table 1:** Mendelian Disorders that Exhibit Microcephaly and Growth Retardation Associated with Defective ATR-Pathway Function

Disorder	Mutant Gene	ATR-Dependent Cellular Features
Seckel syndrome	*ATR, PCNT2* and unknown	Defective ATR-dependent G2-M arrest, supernumerary mitotic centrosomes, DFS expression
Nijmegen breakage syndrome	*NBS1*	Defective ATR-dependent G2-M arrest
Fanconi anaemia	*FANC-A,B,C,D1,D2,E,F,G,H,M,J*	Defective ATR-dependent G2-M arrest, DFS expression
MCPH1-dependent Primary Microcephaly	*MCPH1*	Defective ATR-dependent G2-M arrest, supernumerary mitotic centrosomes

**Table 2:** Genomic Disorders that Exhibit Microcephaly and Growth Retardation Associated with Hemizygous Deletions of ATR Pathway Components

Disorder	Chromosome Deletion	*ATR-Pathway Component*	ATR-Dependent Cellular Features
BPES-ATR^+/-^	3q23	*ATR*	Defective ATR-dependent γH2AX formation, Chk1 phosphorylation and G2-M arrest
Isolated Lissencephaly Sequence	17p13.3	*RPA1*	Defective ATR-dependent γH2AX formation, Chk1 phosphorylation and G2-M arrest
Miller-Dieker Lissencephaly Syndrome	17p13.3	*RPA1*	Defective ATR-dependent γH2AX formation, Chk1 phosphorylation and G2-M arrest
Williams-Beuren Syndrome	7q11.23	*RFC2*	Defective ATR-dependent G2-M arrest
